# The Indirect Genetic Effect Interaction Coefficient *ψ*: Theoretically Essential and Empirically Neglected

**DOI:** 10.1093/jhered/esab056

**Published:** 2021-11-01

**Authors:** Nathan W Bailey, Camille Desjonquères

**Affiliations:** Centre for Biological Diversity, School of Biology, University of St Andrews, St Andrews, Fife KY16 9TH, UK

**Keywords:** indirect genetic effect, interacting phenotype, interaction coefficient, social evolution, trait-based analysis, variance partitioning, quantitative genetics

## Abstract

The interaction effect coefficient *ψ* has been a much-discussed, fundamental parameter of indirect genetic effect (IGE) models since its formal mathematical description in 1997. The coefficient simultaneously describes the form of changes in trait expression caused by genes in the social environment and predicts the evolutionary consequences of those IGEs. Here, we report a striking mismatch between theoretical emphasis on *ψ* and its usage in empirical studies. Surveying all IGE research, we find that the coefficient *ψ* has not been equivalently conceptualized across studies. Several issues related to its proper empirical measurement have recently been raised, and these may severely distort interpretations about the evolutionary consequences of IGEs. We provide practical advice on avoiding such pitfalls. The majority of empirical IGE studies use an alternative variance-partitioning approach rooted in well-established statistical quantitative genetics, but several hundred estimates of *ψ* (from 15 studies) have been published. A significant majority are positive. In addition, IGEs with feedback, that is, involving the same trait in both interacting partners, are far more likely to be positive and of greater magnitude. Although potentially challenging to measure without bias, *ψ* has critically-developed theoretical underpinnings that provide unique advantages for empirical work. We advocate for a shift in perspective for empirical work, from *ψ* as a description of IGEs, to *ψ* as a robust predictor of evolutionary change. Approaches that “run evolution forward” can take advantage of *ψ* to provide falsifiable predictions about specific trait interactions, providing much-needed insight into the evolutionary consequences of IGEs.

## The Interaction Coefficient ψ: Theoretical Importance vs. Empirical Evidence

The interaction coefficient *ψ* is a central, much-discussed parameter of indirect genetic effect (IGE) models. This class of models describes the evolutionary consequences of social environments and is rooted in conceptual and mathematical descriptions of the process by which genes expressed in socially interacting individuals can affect the expression of their partners’ phenotypes (Moore et al. 1997). Such effects can cause evolutionary feedback, and modeling the feedback changes our perspective about the genetics of an enormous variety of interacting phenotypes. Considering IGEs that arise from social interactions also provides clarified detail about the action of selection that then acts upon such traits, informing how they are then predicted to respond to selection. IGE theory uses a quantitative genetic approach that combines and formalizes arguments about social dynamics that have been made in other contexts and subdisciplines of evolutionary biology, such as Griffing’s (1967, 1968a, 1968b) concept of *associate effects*, West-Eberhard’s (1979, 1983) verbal arguments about the evolutionary consequences of *social selection*, Dawkins’ (1982) concept of *extended phenotypes*, and *maternal effects* theory (e.g., Mousseau and Fox 1998). Since its inception, the interacting phenotype framework has been used to detect and characterize IGEs for a wide variety of traits and contexts, such as behavior (reviewed in Bailey et al. 2017), agriculture (reviewed in Bijma 2014), ecology (e.g., Shuster et al. 2006; Mutic and Wolf 2007; Genung et al. 2011; Wolf et al. 2011; Riedel et al. 2018), and medicine (e.g., Baud et al. 2017, 2020).

In these and other research contexts focusing on the effects of IGEs, the interaction coefficient *ψ* is of significant practical importance because it broadly indicates the direction and magnitude of IGEs on specific traits identified by the experimenter. But what is *ψ*? We discuss the biological meaning of *ψ* and raise the issue that several important assumptions made in theoretical models are likely to be violated in empirical studies that estimate *ψ*. In many cases, confounded or biased estimates of *ψ* risk overinflating the apparent magnitude of IGEs, but in other cases, it can be highly illuminating to relax assumptions about *ψ*. We then survey published estimates of *ψ*. Although *ψ* is a central component of most theoretical IGE models that have been published in the nearly quarter-century since the interacting phenotype framework was mathematically formalized by Moore et al. (1997), we identify a considerable gap between this theoretical emphasis and efforts to estimate *ψ* empirically. Although few studies have estimated it, these studies report hundreds of estimates of *ψ*. Further examination reveals heterogeneity in methods used to estimate ψ and its multivariate counterpart, the matrix **Ψ**. However, interesting trends are evident in published estimates of *ψ*, which suggest, though do not firmly establish, that general principles may govern the strength and magnitude of different types of IGEs. Finally, we assert that an important attribute of the interaction coefficient *ψ* has not been capitalized on in empirical studies of IGEs, and that is its power as a predictor of evolutionary dynamics for specified sets of interacting phenotypes. Thus far, *ψ* has almost exclusively been treated as an output of experimental work describing IGEs. We advocate running IGE experiments forward: *ψ* can be treated as powerful input to set predictions about the evolution of specific interacting traits important to basic, agricultural, or medical applications.

## What Is *ψ*?

IGEs occur when genes expressed by one organism affect the expression of traits in a conspecific—such effects are indirect in that genes have a causal effect on phenotypic expression in focal individuals, while not actually residing in those individuals (Moore et al. 1997). This insight can change our view of environmental effects on trait expression and evolution. The environment an individual organism experiences throughout its life cycle can consist of nongenetic features, such as a particular temperature or daylight regime, but provided individuals have social interactions with conspecifics at some point, the environment will also consist of the expressed phenotypes of genes that those individuals carry. If the genes in the environment are variable and cause IGEs, the environmental component of trait expression can itself can be heritable and evolve (Wolf et al. 1999; Bijma 2010; McGlothlin et al. 2010). Understanding this evolutionary feedback between social environments and direct genetic effects (DGEs) can change our perspective of how interacting phenotypes such as aggression, dominance, reproductive behavior, and others evolve (Wolf et al. 1998; Bleakley et al. 2010; Bailey 2012; Bijma 2014).

IGE theory has developed around 2 frameworks that are conceptually different takes on the same phenomenon, but can be mathematically reconciled (McGlothlin and Brodie 2009; Bijma 2014). Variance-partitioning approaches estimate phenotypic variance associated with IGEs, assigning genetic influences on target phenotypes to interacting individuals present in the social environment and quantifying their magnitude relative to DGEs and other sources of environmental variation. The second is a trait-based approach, which focuses on IGEs that are mediated through the expression of traits identified by the experimenter. In trait-based IGE models, the parameter *ψ* is a path coefficient that reveals relationships between these specific traits of focal and interacting individuals (Moore et al. 1997; Bleakley et al. 2010; Bijma 2014; Bailey et al. 2017). It is functionally equivalent to the maternal effect coefficient *m*, and like *m*, *ψ* is assumed to represent a causal relationship (Lande and Price 1989). The interaction coefficient broadly describes how social environments change trait expression within generations. When empirically measured using standardized phenotypic data, it scales between [−1,1] to describe the direction and magnitude of effects of one trait upon another (or upon itself in the case of reciprocal IGEs with feedback, i.e. in which the same trait is involved in both focal and partner individuals). In a univariate example, the effect of trait *j* in a partner on trait *i* in a focal individual with whom they interact would be represented $\psi_{ij}$. The coefficient *ψ*, its multivariate analogue, the matrix ψ, and path diagrams describing the underlying effects they represent have been extensively defined and described elsewhere. Box 1 provides an overview of the biological meaning of the interaction coefficient and its empirical measurement.

Box 1: **The meaning of *ψ***

Previous work provides comprehensive guidance for researchers wishing to empirically quantify IGEs by measuring the interaction coefficient *ψ* (between two traits) or the matrix Ψ of pairwise interaction coefficients between *n* traits where $\Psi_{n,n}=\left(\begin{array}{lll} \psi_{1,1\;} & \cdots & {\;\psi }_{1,n}\\ \vdots & \ddots & \vdots \\ \psi_{n,1\;} & \cdots & {\;\psi }_{n,n} \end{array}\right)$. For technical detail, we refer readers to Moore et al. (1997), Bleakley et al. (2010), McGlothlin and Brodie (2009), Bijma (2010), Bijma (2014), and Bailey et al. (2017). Here we highlight several important conceptual issues that researchers undertaking empirical assessment of IGEs using a trait-based approach may encounter when designing experiments and interpreting *ψ* estimates.

**Figure 1.**
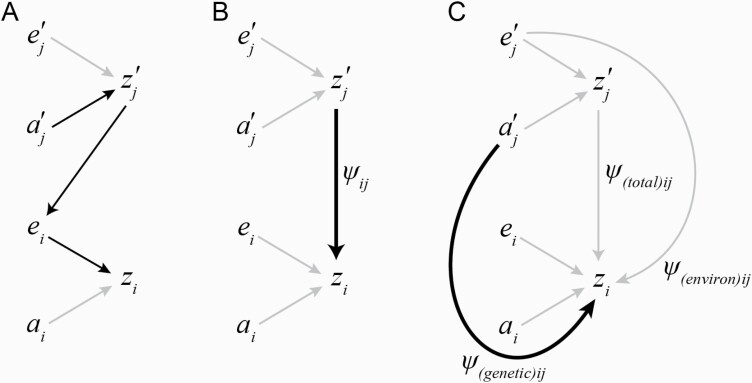
Path diagrams illustrating the components of *ψ* and their relationship to IGEs. Detailed explanations are provided in the Box text. (A) Causal path diagram of an IGE: genes in interacting individuals influence expression of interacting partners’ phenotypes, interacting partner’s phenotypes are a component of the environment of focal individuals; thus, indirect genetic effects influence focal trait expression (after Wolf et al. 1998). (B) The phenotypic association between interacting and focal partner phenotypes is commonly understood to represent *ψ* (after Moore et al. 1997). (C) Typical measurements of the interaction coefficient do not distinguish effects arising from additive genetic versus environmental components of the interacting partner trait, but attempt to control this by eliminating or randomly distributing environmental effects while systematically varying additive genetic effects. However, environmental effects may not be randomly distributed or independent from the focal phenotype, potentially biasing the estimation of *ψ*.

IGEs and the interaction coefficient describing them can be thought of in more than one way. The frequently published path diagrams in Figure 1 illustrate some of these differences. Figure 1A is reproduced from Wolf et al. (1998) and describes causal influences on trait expression in a quantitative genetic framework. The path diagram depicts how IGEs influence focal individual trait expression, $z_{i}$. Arrows in the diagram represent one possible quantitative genetic partitioning of focal trait variation; here showing direct genetic effects $a_{i}$, and all environmental effects, $e_{i}$, influencing focal trait $z_{i}$. A different trait, subscripted *j*, of an interacting partner, represented by the prime, can be considered part of the focal individual’s environment (arrow from $z_{j}^{\prime }$ to $e_{i}$). If some portion of variance in the expression of $z_{i}$ is attributable to the social environment arising from $z_{j}^{\prime }$, then the potential for IGEs exists but is not guaranteed. IGEs only arise from the interacting partner additive genetic effects $a_{j}^{\prime }$. In the case illustrated, it can be seen that the focal individual’s environment contains genes expressed by an interacting individual that exert an influence on focal trait expression. These genetic effects transmitted through social environments are IGEs and are represented by the path of black arrows.

Figure 1B shows how IGEs are commonly measured using the interaction coefficient in trait-based frameworks, and is reproduced from the original description in Moore et al. (1997). This path diagram represents a population-level decomposition of focal trait variance, and as has been described previously, the interaction coefficient $\psi_{ij}$ describes the effect of trait $z_{j}^{\prime }$ in interacting individuals on the value of $z_{i}$ that is expressed by focal individuals. It is a path coefficient analogous to the maternal effect coefficient *m* (Lande and Price 1989; Falconer and MacKay 1996), which is obtained from the partial regression coefficient of $z_{i}$ on $z_{j}^{\prime }$ in a model where data are standardized prior to entry. Thus $\psi_{ij}$ ranges from [−1,1] and describes the magnitude and direction of IGEs involving the two traits.

Figure 1C illustrates an important issue about environmental confounds when empirically estimating *ψ*. IGE approaches assume that environmental effects are randomly distributed with a mean of zero, which can be violated during the empirical measurement of *ψ* due to experimental conditions. That is, if average environmental input into partner trait values, $e_{j}^{\prime }$, is nonzero or genetically or phenotypically correlated the focal trait, then the method used to estimate *ψ* will tend to inflate IGE estimates unless environmental effects oppose the direction of IGEs (Signor et al. 2017a). This is a common problem in any quantitative genetic study, and for that reason early advice on the measurement of *ψ* advised researchers to manipulate genotypic variation in interacting partners using a panel of inbred lines or strains, and phenotype focal individuals of a fixed genotype against these (Bleakley et al. 2010). Such a procedure allows for environmentally unbiased estimation of $\psi_{ij}$ as represented by the black arrow in Figure 1C.

A particularly suitable way to eliminate the possibility of such confounds may be available in systems where the genetic basis of a specific interacting trait is already understood. Experimental treatments that isolate and manipulate the trait in question to measure phenotypic responses in focal individuals can yield robust estimates of *ψ*. Examples include work that experimentally manipulated acoustic signals perceived by field crickets, where the presence or absence of the signal in nature is known to be controlled by a naturally segregating variant (Bailey and Zuk 2012; Pascoal et al. 2016). Technical innovations in other systems might facilitate such an approach when information about the genetic basis of traits is known or estimable, and include techniques such as video behavioral playbacks in fish such as the guppy *Poecilia reticulata* (Woo and Rieucau 2011), electromechanically-controlled sexual signaling behavior in robotic neotropical frogs (*Crossodactylus schmidti*) (Caldart et al. 2020), robotic sage grouse (*Centrocercus urophasianus*) in which variation in female sexual receptivity can be simulated (Perry et al. 2019), or experimentally manipulated body marks (Velando et al. 2013).

A less well-studied but equally important feature of *ψ* is that it provides predictive information about likely evolutionary impacts of IGEs across generations (Moore et al. 1997; Wolf et al. 1998; Bleakley et al. 2010). In the time since Moore et al. (1997) first described the interaction coefficient in IGE models, *ψ* has been persistently emphasized in IGE theory as a fundamental, measurable parameter that describes evolutionary effects of IGEs. Such trait-based models range from the original theoretical work describing evolutionary effects of IGEs (Moore et al. 1997; Wolf et al. 1998; Wolf et al. 1999), to metapopulation models (Agrawal et al. 2001), sexual conflict models (Moore and Pizzari 2005), sexual selection models (Bailey and Moore 2012), social selection models (Wolf et al. 1999; McGlothlin et al. 2010; Bailey and Kölliker 2019; Bailey et al. 2021), game theory models (McGlothlin et al. 2021), models of maladaptation (McGlothlin and Fisher 2021), and models examining the evolution of *ψ* itself (Kazancıoğlu et al. 2012). In each case, evolutionary predictions about the effects of IGEs are either explicitly articulated using the interaction coefficient *ψ* (or matrix **Ψ**), or implicitly dependent on *ψ*. As a general principle, the magnitude and sign of *ψ* in such cases determine the effects that IGEs have on evolutionary outcomes in various scenarios. This predictive property of the interaction coefficient arises from its integral and general role in modifying evolutionary responses to selection in the original trait-based models of Moore et al. (1997) (e.g. Eqn. (8) in Moore et al. 1997 and Eqn. (6) in Chenoweth and Blows 2006). An advantage of this theoretical interest is that many of the underlying assumptions that need to be satisfied to accurately measure and interpret *ψ* have been probed in considerable depth.

## Perils (and Advantages) of Relaxing Assumptions When Measuring *ψ*

A number of important technical assumptions about the measurement of *ψ* are likely to be violated in empirical studies that attempt to measure it. Such risks of incorrect measurement and interpretation are inherent to any empirical work that tests assumptions or predictions of theoretical models, as the latter are reductive by design and living study systems are complex and difficult to control. In recent years, however, some of the assumptions of trait-based IGE models have been usefully characterized and corrected on a theoretical basis, which can help to better align theoretical predictions with empirical evidence about the evolutionary consequences of IGEs.

### Assumption: Nonsocial Environments Can Be Ignored. (They Cannot.)

Several assumptions about measuring *ψ* require eliminating confounds that arise from the nongenetic environments in which individuals develop or interact. Nongenetic environmental contributions to trait expression in both focal and interacting individuals are assumed to be randomly distributed with a mean of zero and uncorrelated (Box 1), yet this assumption may be frequently violated in empirical studies. The first formal resolution to the consequences of such a violation was by Bijma (2014), who proposed a simple analytical correction applicable when *ψ* is measured for IGEs with feedback arising because the IGE involves the same trait in 2 different individuals. An example is aggression. The aggressiveness of a focal individual, $z_{a}$, and that of an interacting partner with whom they are being aggressive, $z\prime_{a}$, cannot be expressed and measured in each individual separately; the phenotypic value for either individual is an emergent property of their interaction. Thus, $\psi_{aa}$ indicates the direction and magnitude of reciprocal IGEs on aggression. In multivariate scenarios where numerous interacting traits are measured, the diagonals of the matrix **Ψ** thus represent reciprocal IGEs with such feedback effects involving the same trait in 2 different individuals (hereafter “on-diagonal IGEs”), and off-diagonals of the matrix represent IGEs involving 2 different traits in respective interactants (hereafter “off-diagonal IGEs”). Note that off-diagonal IGEs may also involve feedback if trait *i* affects trait *j* and trait *j* affects trait *i*. The measurement problem arises because of environmental covariance between focal and interacting partners. In all cases of on-diagonal IGEs, but especially for IGEs of intermediate magnitude, this confounding environmental effect inflates the magnitude of IGE estimates indicated by *ψ* (Bijma 2014). A simple correction has been proposed, hereafter the “Bijma correction,” $\psi_{\left(corr\right)}$, for on-diagonal IGEs:


$$\begin{array}{l} \psi_{\left(corr\right)}=\frac{1-\sqrt{1-b_{P_{i},P_{j}}^{2}}}{b_{P_{i},P_{j}}} \end{array}$$

[Eqn. (12), Bijma 2014]

Here, $b_{P_{i},P_{j}}$ is the partial regression coefficient from a linear model of focal on partner traits and is assumed to be nonzero (Bijma 2014). Of the 238 *ψ* estimates that we found in our literature review (see below), 68 estimates concerned on-diagonal IGEs. However, of the 55 on-diagonal *ψ* estimates published after Bijma (2014) identified this issue, only 6 (ca. 11%) report values with the correction. When comparing the value of all reported on-diagonal IGEs without the correction (back-calculated if the correction was applied originally) versus with the correction (calculated if the correction had not been applied originally), it is clear that the Bijma correction yields a far more conservative overall estimate of on-diagonal IGEs: ${\bar{\psi }}_{\left(uncorr\right)}=0.45$, whereas ${\bar{\psi }}_{\left(corr\right)}=0.28$. This suggests considerable inflation of the magnitude of on-diagonal IGEs exists in the currently published literature. This important correction has thus been applied to extremely few relevant estimates of *ψ*, but it can substantially change interpretations about the consequences of IGEs by making evolutionary predictions about interacting traits with feedback markedly more conservative.

For example, in Rebar et al. (2020), *ψ* was estimated for an important larval fitness trait, mass, in the burying beetle *Nicrophorus vespilloides*. Estimates of IGEs with feedback on larval mass using experimentally evolved focal larvae and sibling larvae with whom they shared a mouse carcass to feed upon. The focal and interacting larvae had evolutionary histories of parental care versus no parental care. The only larval trait measured was larval mass; a reciprocal IGE with feedback as the mass of focal larvae on a carcass could be reasonably expected to be influenced by the mass of its interacting partners on the same carcass. The Bijma correction was applied. For example, when both focal larvae and their carcass-mates had an evolutionary history lacking parental care, the interaction coefficient was positive: ψ=0.553 and $\psi_{\left(corr\right)}=0.302$. This meant that focal and interacting larvae without an evolutionary history of parental care showed mutually reinforcing patterns of weight gain during development. Large focal larvae were associated with large partner larvae and vice versa. This pattern was consistent with greater levels of cooperative sibling behavior having evolved in the absence of parental care, enabling both focal and partner larvae to better exploit their food resource together (Rebar et al. 2020). In contrast, when focal larvae evolved without parental care but their interacting partners evolved with parental care, the sign of the relationship was reversed and ψ=−0.430 whereas $\psi_{\left(corr\right)}=-0.226$. This negative IGE meant that focal and sibling larvae showed antagonistic patterns of weight gain during development, implying a competitive interaction (Rebar et al. 2020). The change in predicted evolutionary response to selection was not insignificant. From Eqn. (16b) (or corresponding Figure 3) of Moore et al. (1997), there was an approximately 2-fold decrease in the relative change of phenotypic evolution predicted by the IGE in conditions where both interacting partners evolved without parental care. Correcting estimates of *ψ* provided a more conservative, though still compelling, prediction about the contribution of reciprocal IGEs on larval mass to evolutionary transitions between sibling rivalry and parental care. In general, inflated estimates of *ψ* severely impact evolutionary inference about the role of IGEs. This is particularly true for large absolute values of *ψ*, because relative change in phenotypic evolution does not scale linearly with change in the interaction coefficient. Rather, it can approach infinity as *ψ* approaches its maximum absolute magnitude (Moore et al. 1997; McGlothlin et al. 2010).

There is another method of estimating IGEs that circumvents the problem of inflated on-diagonal IGEs. McGlothlin and Brodie (2009) developed a multivariate, pedigree-based approach to estimate **Ψ** using the direct additive genetic variance component $G_{D}$, the social additive genetic variance component $G_{S}$, and their covariance $G_{SD}$ which for traits of interest:


$$\begin{array}{l} \Psi =G_{SD}G_{D}^{-1} \end{array}$$

[Eqn. (14), McGlothlin and Brodie 2009]

**Figure 2.**
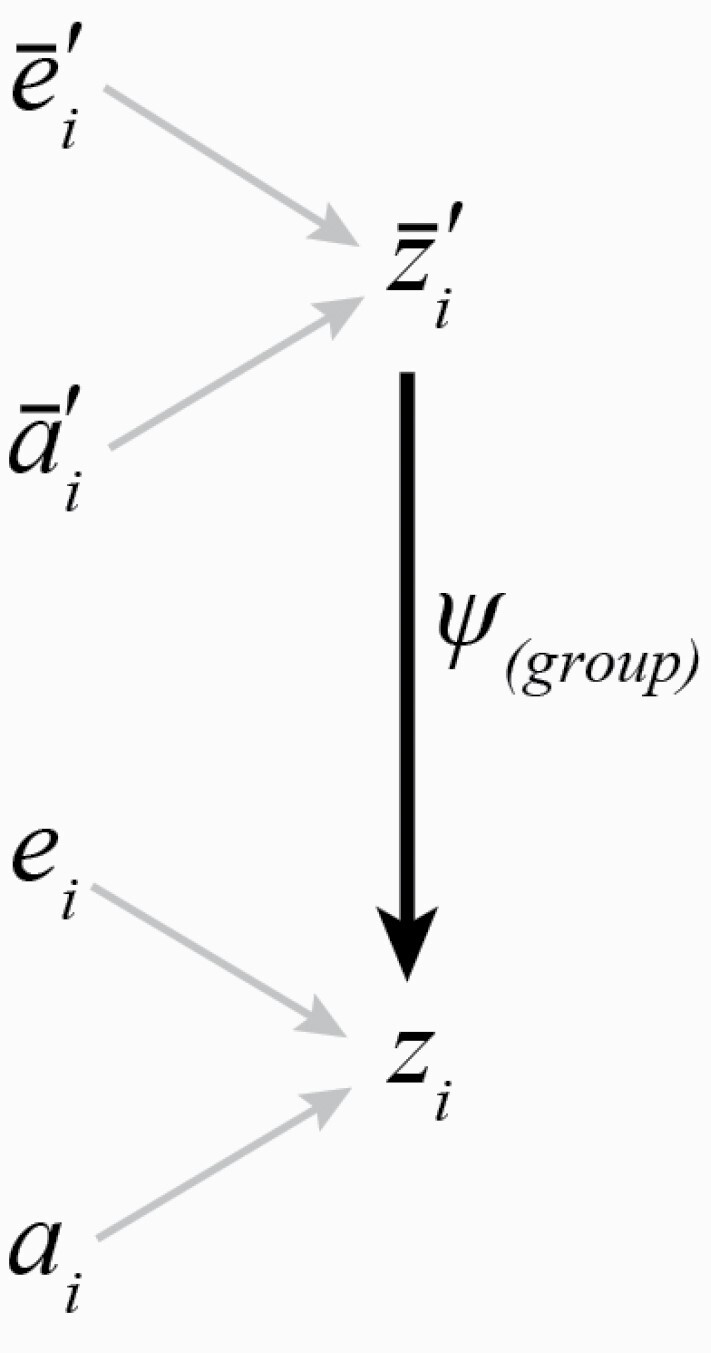
When a focal phenotype is influenced by interacting phenotypes of more than one individual, $\psi_{\left(group\right)}$ can be conceived as the mean of IGEs arising from all interactants in the group (McGlothlin and Brodie 2009).

The relationship linking **Ψ** to variance components provides a powerful method for converting commonly estimated quantities in variance-partitioning experiments to *ψ* estimates which can then be compared systematically across different contexts or studies to gain broader insight into the prevalence and characteristics of IGEs. In this case, the social additive genetic variance component $G_{S}$ represents the heritable component of social effects on an individual’s phenotype (Griffing 1967; Bijma et al. 2007; McGlothlin and Brodie 2009). This method has the added benefit of not requiring the Bijma correction because feedback affects $G_{SD}$ and $G_{D}$ equally, and they thus cancel one another. Although the approach has not yet been used to calculate ψ, it has been used to estimate the analogous matrix of maternal effect coefficients **M** in the American Bellflower, *Campanula americana* (Galloway et al. 2009), suggesting feasibility for a wide range of IGE studies. An additional advantage is that this technique is extendable to measuring group-level IGEs, that is, IGEs arising not only from the interaction between a focal individual $z_{i}$ and an interacting partner ${z^{\prime }}_{j}$, but also the effects of the interacting partner’s social interactions ${z^{\prime \prime }}_{j}$. To capture group IGE dynamics, it is possible to estimate an average, group-level interaction coefficient $\psi_{\left(group\right)}$ (Figure 2) (McGlothlin and Brodie 2009). Such an approach may permit a more realistic understanding of interacting partner effects on focal trait expression, for example allowing integration of effects across successive social encounters (Schneider et al. 2017). Another method to evaluate group effects was proposed by Saltz (2013) and tests how the interaction between two individuals is affected by the presence of a third.

A shared nongenetic environment of focal and interacting individuals could also confound measurement of *ψ* in cases where *different* traits in focal and partner individuals are measured. For example, if an experimenter sought to estimate *ψ* by manipulating the genotype of interacting partners, shared rearing or other environmental experience could inflate the apparent relationship of any traits measured in the two individuals (see Box 1, Figure 1C). Such a situation might arise if, for example, test subjects of a laboratory study species were paired within test tubes or 96-well plates; the physical environment might differ from tube to tube or well to well, and this shared nonsocial environment could be expected to enhance any apparent phenotypic association between focal and interacting individuals (Marie-Orleach et al. 2017; Signor et al. 2017a, 2017b).

In many systems, this problem can be overcome by maintaining individuals in a common lab environment until the point of social interaction and phenotyping, which then occurs in a standardized and controlled setting. Such a procedure would minimize shared effects of the nongenetic environment. In other experiments, developmental IGEs might be desirable to estimate, in which case a blocked and randomized design is required to account for variation associated with shared nongenetic environments. In the burying beetle example above, the effects of a shared focal and partner environment are perhaps conceptually easier to perceive because multiple focal and interacting larvae were reared on a single mouse carcass (*N. vespilloides* larvae feed on dead flesh) and their fitness phenotypes measured (Rebar et al. 2020). Thus, in addition to correcting *ψ* for traits with feedback, there was a shared local environment of focal and interacting partners that required controlling through randomized pairings of focal and interacting individuals across multiple carcass replicates (Rebar et al. 2020).

### Assumption: *ψ* Is Fixed. (It Is Not.)

Despite ample countervailing evidence, *ψ* is almost always assumed for convenience to be a fixed, population-level parameter in trait-based IGE models. However, one of the first and arguably most prominent insights from empirical research estimating the interaction coefficient is that it is frequently not fixed (Table 1). Such studies have firmly established the genetic variability of *ψ*, and thus its potential responsiveness to selection, as a general principle. How has this been demonstrated? In the trait-based IGE framework, genotype-by-genotype IGEs (GxG IGEs, or GxG epistasis) occur when *ψ* varies by strain or genotype. GxG IGEs are also readily detected using variance-partitioning approaches (e.g. Rode et al. 2017). GxG IGEs have been found directly, or strongly inferred through the detection of population-level variation in *ψ*, in the fruit fly *Drosophila melanogaster* (Kent et al. 2008), the field cricket *Teleogryllus oceanicus* (Bailey and Zuk 2012), the flatworm *Microstomum lignano* (Marie-Orleach et al. 2017), the guppy *Poecilia reticulata* (Bleakley and Brodie 2009; Edenbrow et al. 2017), and the mosquitofish *Gambusia holbrooki* (Kraft et al. 2018; Culumber et al. 2018). In addition to measuring strain-specific *ψ*, there have been estimates of sex-specific *ψ* (Edenbrow et al. 2017) and even environment-specific *ψ* (Signor et al. 2017a).

**Table 1.** Summary of articles estimating *ψ* using a trait-based approach

Article	Experimental subject	Focal trait type (exact traits)	Partner trait type (exact traits)	No. of *ψ* estimates	*ψ* range^a^ and SD^b^	Other variable^c^
Bleakley and Brodie (2009)	Guppy (*Poecilia reticulata*)	Behavior (time in proximity, time oriented, time agitated, time schooling, inspection)	Behavior (proximity time, time oriented, time agitated, time schooling, inspection)	65	−1.14 to 0.93 (0.388)	Lines
Bailey and Zuk (2012)	Field cricket (*Teleogryllus oceanicus*)	Behavior and morphology (mounting latency and mass)	Behavior (song/no song)	12	−0.627 to 0.402 (0.32)	Population, generation
Bleakley et al. (2013)	Isopod (*Thermosphaeroma thermophilum*)	Behavior (latency to cannibalize)	Morphology (relative body size)	1	−0.048	None
Saltz (2013)	Fruit fly (*Drosophila melanogaster*)	Behavior (lunge number)	Behavior (aggression)	1	0.101	Lines
Bailey and Hoskins (2014)	Fruit fly (*D. melanogaster*)	Behavior (tapping behavior)	Behavior and survival (chill coma, MSB and paraquat survival, startle response, starvation resistance, orienting, following, tapping, licking, singing, mounting, general activity)	12	−0.696 to 1.299 (0.521)	None
Han et al. (2016)	Water strider (*Gerris lacustris*)	Behavior (same sex behavior)	Behavior and morphology (same sex behavior and body size)	2	−0.130 to 0.001 (0.093)	None
Pascoal et al. (2016)	Cricket (*Teleogryllus oceanicus)*	Chemical (cuticular hydrocarbon)	Behavior (song/no song)	6	−0.45 to 0.39 (0.335)	None
Anderson et al. (2017)	Fruit fly (*D. melanogaster*)	Behavior (aggregation)	Behavior (aggregation)	1	0.042	None
Edenbrow et al. (2017)	Guppy (*Poecilia reticulata*)	Behavior (distance and time)	Behavior (distance, time, and coordination)	80	−0.3 to 2.0 (0.550)	Population, sex, predation
Marie-Orleach et al. (2017)	Flatworm (*Macrostomum lignano*)	Morphology (body size, testis size, ovary size, seminal vesicle size)	Morphology (body size, testis size, ovary size, seminal vesicle size)	16	−0.070 to 0.323 (0.114)	Lines
Signor et al. (2017a)	Fruit fly (*D. melanogaster* and *simulans*)	Behavior (movement rate)	Behavior (movement rate)	28	0.04 to 0.960 (0.236)	Species, line, ethanol
Signor et al. (2017b)	Fruit fly (*D. melanogaster*)	Behavior (movement rate)	Behavior (movement rate)	12	0.16 to 0.54 (0.110)	Line, ethanol
Culumber et al. (2018)	Mosquitofish (*Gambusia holbrooki*)	Behavior and morphology (feeding, length, mass, and condition)	Morphology (color)	4	0.035 to 0.960 (0.438)	Lines
Kraft et al. (2018)	Mosquitofish (*Gambusia holbrooki*)	Behavior (hiding and principal components)	Morphology (color)	6	−0.375 to 0.20 (0.221)	Lines
Rebar et al. (2020)	Burying beetle (*Nicrophorus vespilloides*)	Morphology (body mass)	Morphology (body mass)	4	−0.430 to 0.722 (0.293)	Lines

^a^See main text for explanation of absolute values exceeding 1.00.

^b^Standard deviation of all *ψ* estimates within each study; not indicated for studies presenting only one estimate.

^c^Some studies assessed variation in *ψ* across different contexts (e.g., ecological) or for different genotypes (e.g., across laboratory lines or strains: G × G epistasis or a G × G IGE).

The sister species *D. melanogaster* and *D. simulans* provide an illustrative example of the complexity such interactions can involve. In *D. melanogaster* exposed to ethanol, *ψ* for locomotion varied across different genotypes, but in the same species under control environments without ethanol, it did not (Signor et al. 2017a). By contrast, *ψ* for locomotion was uniform in all environments experienced by *D. simulans* (Signor et al. 2017a, 2017b). Thus, the direction of locomotion IGEs was similar across closely related species, but there appears to be an evolved difference in the environmental sensitivity of those IGEs. Other studies provide more direct evidence that *ψ* evolves. Work on cuticular hydrocarbon composition in *Drosophila serrata* demonstrated experimental evolution of *ψ* (Chenoweth et al. 2010); it was shown to reverse sign across two time points in the same population of the field cricket, *T. oceanicus* (Bailey and Zuk 2012), and the burying beetle *N. vespilloides* evolved divergent signs and magnitudes of *ψ* after 22 generations of laboratory experimental evolution starting from an admixed population (Rebar et al. 2020).

Usefully, Kazancıoğlu et al. (2012) relaxed the assumption of *ψ* as fixed and modeled it as a genetically variable trait partitioned into additive genetic $\left(a_{\psi }\right)$ and environmental $\left(e_{\psi }\right)$ components:


$$\begin{array}{l} \psi =a_{\psi }+e_{\psi } \end{array}$$

[from Eqn. (3), Kazancıoğlu et al. 2012]

The authors then derived expected responses to selection of interacting traits in a variety of scenarios involving nonreciprocal and reciprocal IGEs. In general, selection on *ψ* which increases its value tends to enhance the evolutionary effects of corresponding IGEs (Kazancıoğlu et al. 2012). This intuitive result means that average trait values that would already be predicted to evolutionarily increase due to the effects of IGEs will increase faster when *ψ* evolves to become larger, due to compounding feedback effects. However, the authors make the insightful observation that selection on *ψ* can oppose the direction of trait evolution caused by IGEs, severely dampening expected responses to selection (Kazancıoğlu et al. 2012). This means that the direction in which *ψ* is evolving may be as important as its fixed value at any given point in evolutionary time for determining the evolutionary consequences of IGEs. Given the preponderance of evidence that *ψ* is genetically variable and thus responsive to selection, it must be considered that coevolution of interacting traits and *ψ* itself may be of considerable significance in the evolutionary process. In the future, quantitative genetic treatments examining genetic variance in the interaction coefficient *ψ* are likely to contribute greater biological realism to trait-based IGE studies.

### Assumption: There Are No Latent Variables. (There Are.)

Without careful experimental control, it is challenging to eliminate the possibility that an undetected latent variable distorts estimates of IGEs on specific traits. Empirical work using the *Drosophila* Genetic Reference Panel of inbred *D. melanogaster* lines has attempted to detect such “cryptic” IGEs using screening procedures to enter numerous interacting phenotypes as explanatory variables into a multiple regression (Bailey and Hoskins 2014). This approach is constrained by the pool of traits available for phenotyping, but regression analysis at least permits the conditional assessment of potentially correlated interacting traits (Morrissey and Ruxton 2018). Latent variables matter in trait-based approaches because if the objective is to understand evolutionary dynamics caused by IGEs involving particular interacting and focal traits, then it is necessary to minimize the possibility that an IGE involving a different, undetected, interacting trait does not underlie evolutionary dynamics.

## The Magnitude, Sign, and Variability of Published *ψ* Estimates

**Figure 3.**
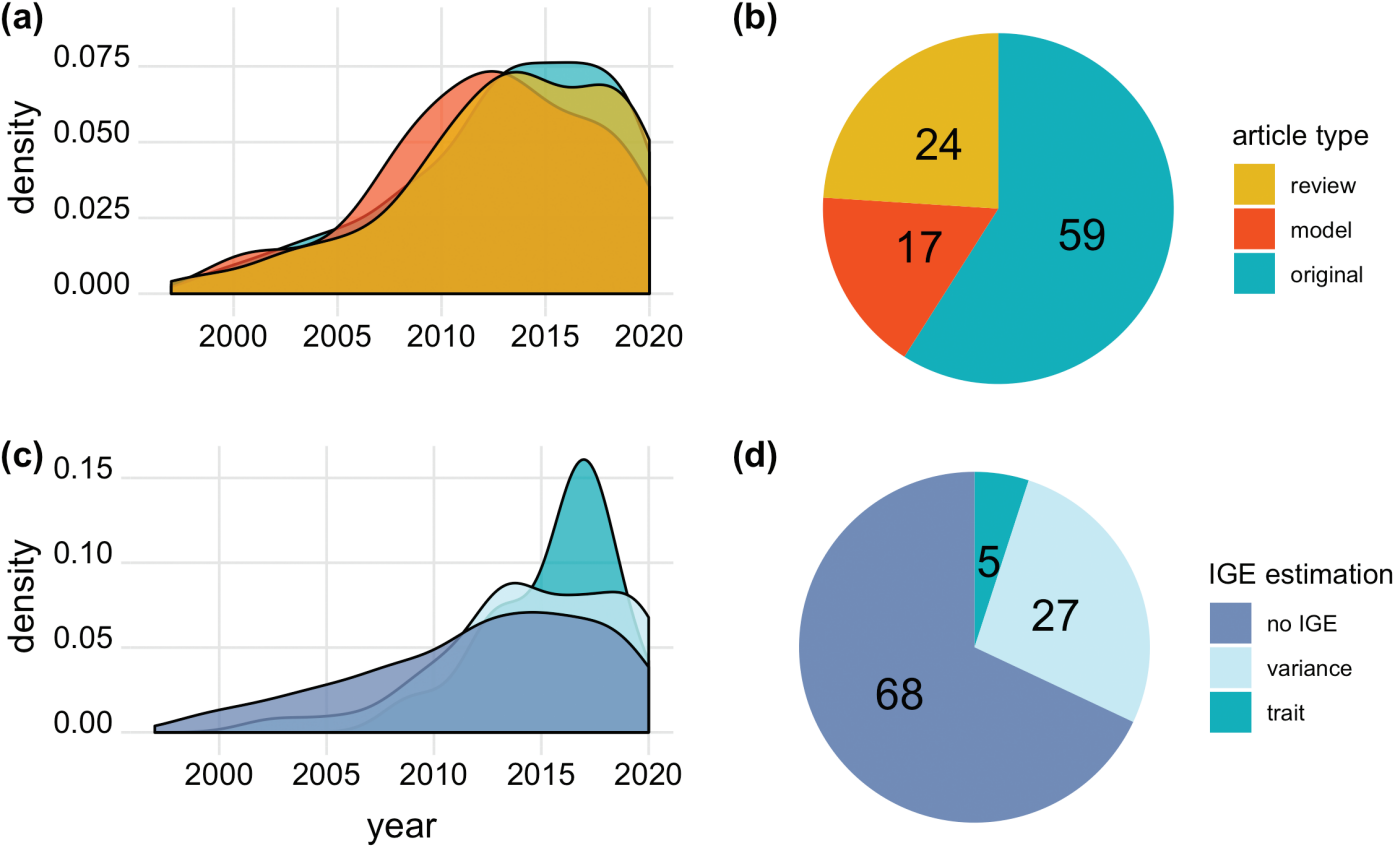
Distributions and types of 463 articles citing Moore et al. (1997), assessed in March 2021. (A) Density distribution of the three different article types per year illustrating relative differences in publications over time. (B) Pie chart showing the percentages of each article type. (C, D) Distributions of the approaches of IGEs estimations in original articles citing Moore et al. (1997) illustrating relative differences in publications over time. (C) Density distribution of the IGE estimation approach per year. (D) Pie chart showing the percentages of each IGEs estimation approach. The year 2021 was removed from the density distributions as it biases distributions towards lower values.

Much research has been focused on the effect of the social environment on behavior, morphology, and physiology. To first gain a retrospective view of studies that have used an IGE framework, we extracted articles that cite Moore et al. (1997) using a Web of Science search in March 2021. We summarized the 463 scientific journal articles citing this original article introducing IGEs, their interpretation, and their measurement using *ψ*, and manually classified these into review articles, original empirical studies, or theoretical models (Supplementary Table S1). Approximately 40% were review and theoretical modeling articles (Figure 3A and B). Among all articles, 275 (59%) presented original data and most were published after 2010, 13 years after the first publication of the IGE framework and introduction of *ψ* as a concept (Figure 3A and B). Less than a third of these original articles (89 of 275, 32%) actually estimated IGEs, and the overwhelming majority of the estimations of IGEs (74 of 89) used a variance-partitioning approach (Figure 3C and D). We found a total of 15 articles estimating IGEs using a trait-based approach. We focused on estimates of *ψ* only and did not consider maternal or paternal effects, as the latter comprise part of a separate literature that has had much more extensive and distinct development.

Although only 15 studies estimated IGEs using trait-based approach, these yielded a total of 238 *ψ* estimates in 9 animal species (1 platyhelminth, 6 arthropods, and 2 ray-finned fish) (Table 1). For the purposes of our enquiry, in cases where authors estimated the matrix **Ψ**, we counted each entry in the matrix as a single estimate of *ψ*. Each article estimated on average 17 values of *ψ* (range: 1–80). The wide majority of focal traits in these articles were behavioral (203 traits), 29 were morphological and 6 were chemical (cuticular hydrocarbon profiles of *T. oceanicus* in Pascoal et al. 2016). Similarly, most of the interacting partner traits were behavioral (205 traits), 31 were morphological, and 2 were survival. The social context in which *ψ* was estimated ranged from courtship and mating, aggression, cannibalism, to cooperation. The variation of *ψ* reported by these articles should be interpreted cautiously, as there was almost complete nonuniformity in the design of experiments to estimate it. All the studies implemented a multiple-regression-type approach, with model variations to account for features of the experimental design such as blocked or crossed factors. What varied, however, was the nature of the predictor variable(s). The interaction coefficient, for example, was estimated through the measurement of focal trait variation assessed against interacting partners from different genetic lines, species, populations and generations (ancestral vs. current) and in response to different experimental conditions such as predation regimes or presence of exogenous agents such as ethanol (Table 1).

Estimates of *ψ* ranged between −1.14 and 2.00 (Table 1). It is worth noting that, as formally defined, the absolute value of *ψ* cannot exceed 1, so empirical measures that exceed this obviously require some explanation. As detailed in Box 1, *ψ* is estimated using partial regression coefficients from a linear model examining the effects of partner trait values on focal trait expression; data are standardized prior to entry in the model. As has been noted previously (Bailey and Hoskins 2014), there can be considerable variation around these model estimates, and when such variability is combined with high absolute values of *ψ*, it is advisable to report exact values obtained but cautiously consider them censored at −1 or 1 for purposes of onward experimentation or biological interpretation. Another potential cause of absolute values of *ψ* that exceed 1 is if trait values have not been standardized to the same scale prior to regression analysis, although this does not appear to be the case in the empirical work that has reported |ψ|>1 (Bleakley and Brodie 2009; Bailey and Hoskins 2014; Edenbrow et al. 2017).

**Figure 4.**
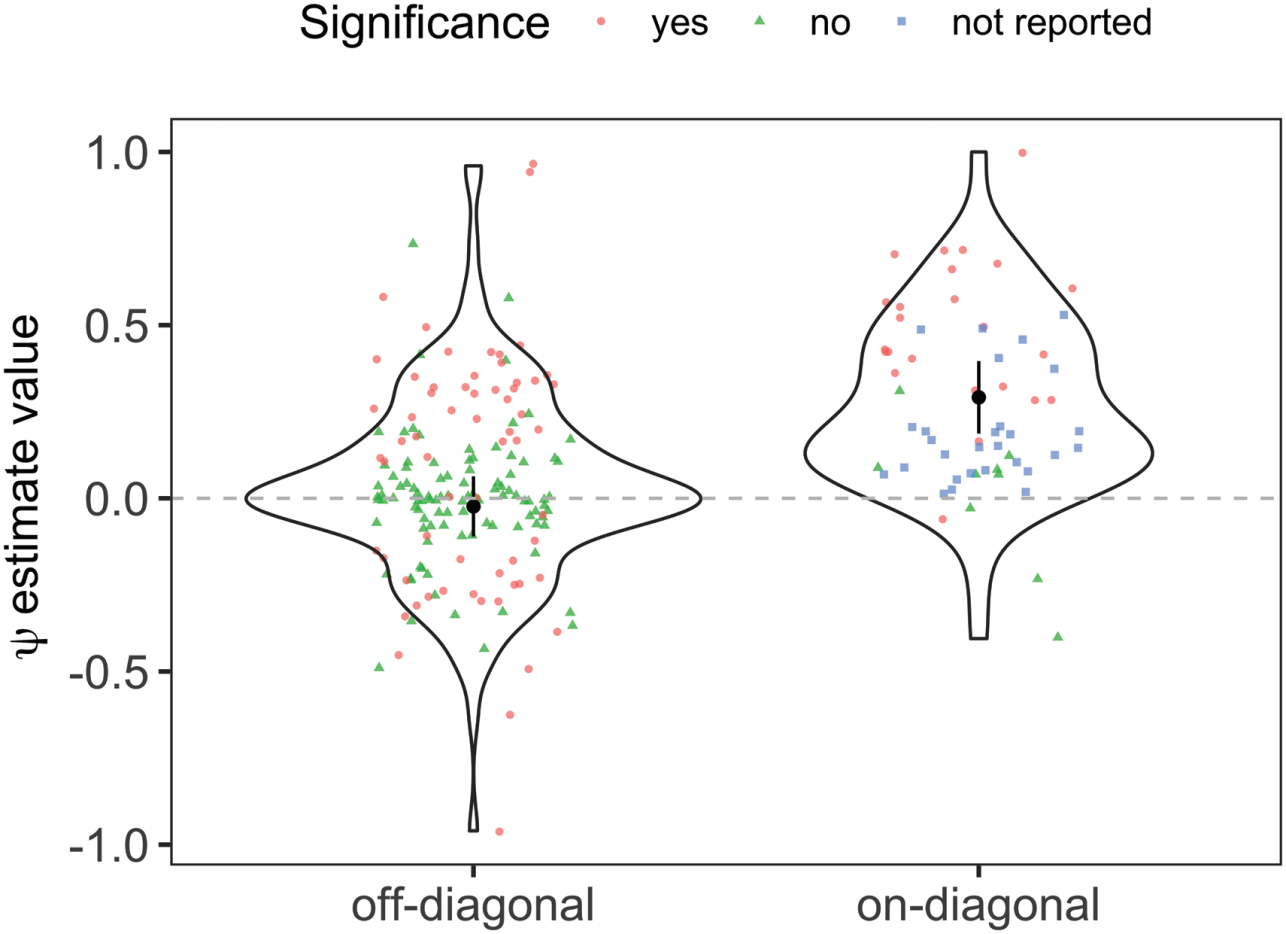
Violin plots showing magnitude of *ψ* estimates for on-diagonals (reciprocal IGEs involving the same trait) versus off-diagonals (IGEs acting on different traits). Points show the 225 estimates of *ψ* analyzed, with significance in the original study indicated with red circles (significant), green triangles (nonsignificant), or blue squares (not reported). The black points and bars show the fitted values and standard error obtained with a linear mixed model including *ψ* as the response variable, IGE type (on-diagonal or not) as a fixed effect and study identity as random factor. Note that all values where |ψ|>1 were removed for this analysis, as it is impossible to estimate $\psi_{\left(corr\right)}$ for these. If these values (*n* = 13) are included in the analysis uncorrected, the outcome is qualitatively the same: the magnitude of reciprocal IGEs with feedback is significantly greater. See the main text for statistical details.

Empirically estimated values of *ψ* were mostly positive, with 148 positive estimates against 70 negative estimates and 20 that were indistinguishable from zero (Figure 4; Table S2). Interestingly, there were significantly more positive values for reciprocal IGEs with feedback, that is, diagonal entries in the matrix **Ψ** involving the same focal and interacting trait which mutually affect one another in interacting partners (generalized linear mixed model with sign of *ψ*—positive or negative—as the response, including *study identity* as a random effect (variance explained: 0.216): χ ^2^ = 27.6, *P* < 0.001). Thus, off-diagonal IGEs, which represent potential IGEs involving 2 different traits, are less likely to be mutually reinforcing in the sense that evolutionary responses attributable to these IGEs would tend to accelerate trait elaboration.

The values of IGEs also varied. We used the Bijma correction on all on-diagonal IGEs reported in the literature (unless it had already been applied) to obtain reliable estimates, and tested the difference in value between on-diagonal and off-diagonal IGEs. For this test, we removed absolute values of *ψ* exceeding 1 because the Bijma correction cannot be applied as it would result in an imaginary number. This necessitated removing 13 values of *ψ* (6 on-diagonal and 7 off-diagonal). There were too few studies reporting estimates of *ψ*, and even fewer reported with their associated error, to warrant a meta-analytical approach, and we examined raw reported values to avoid over- or under-inflating estimates of absolute magnitude (Nakagawa and Lagisz 2016). The resulting analysis was thus unbiased, and revealed that values of *ψ* were significantly higher for on-diagonal IGEs than off-diagonal IGEs (Figure 4) (linear mixed model including *study identity* as a random factor (variance explained: 0.009): χ ^2^ = 43.31, *p*-value < 0.001). Estimates for on-diagonal versus off-diagonal IGEs were 0.292 and −0.023, respectively. In aggregate, these patterns suggest a general tendency for IGEs with feedback to be more prone to strong, mutually augmenting evolutionary responses, but it is impossible to exclude the possibility that the pattern arises from biases in the types of traits studied by researchers. Nevertheless, it is a striking pattern that merits further systematic consideration—are reciprocal IGEs with feedback generally positive and of greater magnitude, and if so, why should that be the case? An intriguing possibility is that there is a general, directional constraint on the responsiveness of trait expression during social interactions, such that it is more likely that an organism flexibly increases expression of a given trait after or during social interaction, than decreasing its expression. Although counterexamples exist, such as can be observed in winner-loser effects during contests, future work would benefit from considering whether escalation, rather than de-escalation, is a more likely outcome across a range of interaction types.

Both across and within studies, *ψ* values were highly variable with a global standard deviation of 0.45 and standard deviations ranging from 0.093 to 0.550 (median = 0.335) within studies. We were able to assess reported statistical significance for 209 values of the interaction coefficient, and almost half of these (99 of 209) were significant at α=0.05 (Figure 4). The statistical significance of these estimates may need to be considered carefully as many articles report several values of *ψ* without controlling for multiple testing. The application of corrections for false discovery rate in evolutionary and behavioral ecology (among other life sciences fields) is not without controversy, and care must be taken to balance a suitably conservative interpretation against the aims of the study (e.g., Moran, 2003). The risk of spurious significance is likely to be heightened in studies that report numerous IGE estimates across multiple conditions, lines, or other contexts. However, where the aim of a study is primarily descriptive or a hypothesis-generating exercise, and the purpose of characterizing IGEs using *ψ* is not to infer evolutionary dynamics that have happened but is instead to discover potential IGEs for later experimental follow-up, it is advisable to consider effect sizes, statistical power, and independently replicate key findings rather than rely on statistical significance as the sole criterion for confidence.

## Making Better Use of *ψ* in Evolutionary, Behavioral, and Ecological Research

The mismatch between theoretical emphasis on the interaction coefficient *ψ* and its empirical measurement in IGE studies is perhaps not surprising given the availability of an alternative approach to measuring IGEs—variance partitioning—which is based on established and widely practiced techniques in statistical quantitative genetics. In this approach, which is explained in further detail in Moore et al. (1997), Bijma (2010, 2014), Bleakley et al. (2010), McGlothlin and Brodie (2009), and Bailey et al. (2017), variance of a particular phenotype is partitioned among DGE, IGE, and other nonheritable effects using standard modeling approaches applied to data from a quantitative genetic experimental design. Evolutionary potential depends on DGEs and IGEs, and critically, their covariance. The fraction of phenotypic variance caused by genes in interacting partners can be estimated irrespective of the traits present in those partners through which IGEs are mediated. Though not a trait-based approach, variance partitioning is by no means “trait free.” This and allied approaches for studying contributions of social environments to expressions of heritability and response to selection such as family-level selection and linear animal models have been developed to elicit targeted improvements in traits of agricultural interest (Ellen et al. 2007; Ellen et al. 2010). Thus, IGEs may simply be more challenging to measure using the interaction coefficient *ψ*; to quote the authors of the original description of the interaction effect coefficient, “It is of interest to evaluate *ψ* for a number of characters. Unfortunately, this is logistically difficult.” (Moore et al. 1997).

The logistical difficulty of estimating *ψ* is one potential cause of the taxonomic bias in our survey of empirical estimates to date, which favor invertebrate laboratory systems in which the necessary manipulation of genetic strains, or plausible substitute approaches, can be achieved. The overrepresentation of such systems may also reflect stochastic bias caused by the relatively small number of studies our survey recovered. Despite such practical challenges, *ψ* captures the interest and imagination of researchers studying the effects of genes in the social environment, with arguably as many versions of its now classical path diagrams published as there have been empirical studies of it. More estimates would be helpful to researchers interested in understanding the general importance and magnitude of IGEs in the evolutionary process. However, we propose that it would also be useful to use estimates of the interaction coefficient in a different manner than has been to date: instead of treating *ψ* solely as an output of experimental work which describes IGEs present in extant populations, strains or species, greater insights may lie in viewing it as an input into evolutionary predictions about the future of traits and processes of interest to researchers in behavior, ecology and evolution.

As a descriptor of IGEs, *ψ* is theoretically well-grounded and intuitive, but as a predictor of evolutionary consequences, its role has been neglected. Experimental work that can manipulate the starting conditions of populations to include varied IGEs would be extremely valuable for testing the significance of IGEs for the evolutionary process, and *ψ* provides an intuitive measure for characterizing those starting conditions and setting out falsifiable predictions. Such an approach is most easily tailored in laboratory experimental evolution systems. Several of the studies we surveyed take advantage of such systems, for example by demonstrating the evolution of IGEs by showing evolved change in *ψ* in *Drosophila serrata* (Chenoweth et al. 2010), or by experimentally manipulating social environments in the burying beetle *Nicrophorus vespilloides* and testing whether *ψ* differed among treatments at the end-point of that process (Rebar et al. 2020). The additional step of characterizing *ψ* initially to set up treatments in which *ψ* varies (Signor et al. 2017b), and then running artificial evolution forward to measure the predicted evolutionary responses for traits of interest, has yet to be taken.

A “running-evolution-forward” approach would be amenable in contexts where interacting phenotypes are of particular interest to experimenters, and *ψ* either has been estimated already or can easily be estimated. The case of sexual selection’s role in reproductive isolation and speciation is illustrative. It is well-known from a variety of taxa that female mate choice, including female preferences for male trait values, can respond to the social environment (Rodríguez et al. 2013; Rebar and Rodríguez 2015; Fowler-Finn et al. 2017; Desjonquères et al. 2021), and it is also theoretically predicted that such flexibility, when underpinned by IGEs, can enhance the opportunity for runaway trait-preference coevolution (Bailey and Moore 2012). It therefore stands to reason that the presence of IGEs could alter the opportunity for speciation to occur as well as its speed. It seems plausible that IGEs could cause a diversity of outcomes, depending on the nature of the selected traits involved (behavioral interactions, communication signals, morphological ornaments, or multimodal displays) and the manner in which receivers detect and respond to them (sensory mode(s), whether flexible preferences are active or passive), and the degree to which *ψ* does or does not vary across allopatric or parapatric populations. Prior work on the role of learning during reinforcement has suggested an impact of learned changes in conspecific versus heterospecific mate discrimination (Servedio et al. 2009; Servedio and Dukas 2013), but IGE theory allows for additional evolutionary and developmental feedbacks driven by the social environment acting as both a cause and target of selection. The coefficient *ψ* provides a framework with which to predict evolutionary consequences depending on unidirectional vs. reciprocal trait interactions and those with vs. without feedback.

Experimental work addressing such issues would be subject to several caveats that are generally applicable to the estimation of *ψ* as described above, such as confounds arising from shared environmental effects. In this case, if different populations or strains were used in experimental work, care would be required to ensure that population-specific estimates of *ψ* reflect genetic, as opposed to environmental, variation (unless environmental effects on IGEs were specifically in question). For these reasons, we envision 2 main “running-evolution-forward” approaches for IGE work. The first is artificial evolution in laboratory conditions, where experimental confounds are more readily controlled or accounted for in experimental design. Microbial and invertebrate systems would appear more suited to such an approach for practical reasons, but we note that IGE studies in the lab increasingly take advantage of genetic resources in vertebrate systems such as inbred mouse lines (*Mus musculus*) and guppies (*Poecilia reticulata*) (Bleakley and Brodie 2009; Baud et al. 2017, 2020). The second approach is to use long-term field study systems. Instead of estimating IGEs and inferring past process, it would be informative to estimate IGEs and predict future outcome. Responses to selection can be predicted using known information about populations with different initial values of *ψ*. There may be relatively few natural systems in which the infrastructure for such long-term data collection is in place, but we expect this situation to improve as the utility of such systems for IGE studies becomes more widely appreciated. Examples include red deer (*Cervus elaphus*) which have been extensively studied in Scotland (Wilson et al. 2011), field crickets (*Teleogryllus oceanicus* and *Gryllus campestris*) which have been monitored in Hawaii and Spain, respectively (Zuk et al. 2018; Rodríguez-Muñoz et al. 2019), and Trinidadian guppies (*Poecilia reticulata*) which have been studied over time in replicate river systems in nature (Ghalambor et al. 2015). As with all consideration of laboratory-based versus field-based research, experimental control and biological realism will trade off to some extent. However, these and other long-term field systems may, when complemented with laboratory work, provide particularly valuable opportunities for testing the evolutionary impacts of IGEs.

The interaction coefficient *ψ* can play a powerful role in setting up clear, falsifiable predictions about traits of interest in experimental approaches. Research estimating the interaction coefficient *ψ* has provided satisfying insights on the evolutionary dynamics of interacting phenotypes at particular, fixed moments. However, evolution is dynamic, and that of social interactions is expected to be particularly dynamic as IGEs can introduce an element of volatility (Moore et al. 1997; McGlothlin et al. 2010; Bailey and Kölliker 2019). Future studies that can track the influence of IGEs as they do, or do not, exert effects on processes such as adaptation, social evolution, and diversification will contribute significantly to our understanding of the origins and maintenance of complex social phenotypes, and by extension, the evolutionary process itself.

## Supplementary Material

esab056_suppl_Supplementary_Table_S1

esab056_suppl_Supplementary_Table_S2

## Data Availability

All data underlying descriptive analyses of IGE publications and empirical estimates of *ψ* are available as online Supplementary Files S1 and S2.
